# Supplementary material for: Conserved fiber topography of the anterior limb of the internal capsule in treatment-resistant psychiatric patients
Source: bioRxiv. 2026 May 15:2026.05.11.724148. Preprint. [Version 1] doi: 10.64898/2026.05.11.724148 (PMC13192871; doi:10.64898/2026.05.11.724148)
Supplement: 1 [file NIHPP2026.05.11.724148V1-supplement-1.pdf]

bioRxiv preprint doi: https://doi.org/10.64898/2026.05.11.724148; this version posted May 15, 2026. The copyright holder has placed this preprint (which was not certified by peer review) in the Public Domain. It is no longer restricted by copyright. Anyone can legally share, reuse, remix, or adapt this material for any purpose without crediting the original authors.

Supplemental Table 1: MNI152 coordinates of lead DBS electrodes in OCD patients

		Left Hemisphere			Right Hemisphere		
Patient ID	VC/VS Contacts	x (mm)	y (mm)	z (mm)	x (mm)	y (mm)	z (mm)
OCD001	Contact 1	-10.47	2.86	-9.36	11.62	5.14	-6.25
	Contact 2	-11.79	4.69	-6.86	13.28	6.81	-3.61
	Contact 3	-13.17	6.54	-4.33	15.06	8.52	-1.12
	Contact 4	-14.61	8.35	-1.87	16.82	10.15	1.14
OCD002	Contact 1	-9.83	0.41	-4.42	8.80	2.79	-8.48
	Contact 2	-11.80	2.106	-1.97	10.62	4.56	-5.83
	Contact 3	-13.69	3.68	0.403	12.41	6.24	-3.23
	Contact 4	-15.54	5.19	2.72	14.25	7.94	-0.72
OCD003	Contact 1	-11.54	7.05	-7.61	9.96	-0.35	-4.62
	Contact 2	-13.16	8.31	-4.61	11.45	1.49	-2.43
	Contact 3	-14.91	8.93	-1.57	12.94	2.75	0.13
	Contact 4	-16.81	8.98	1.55	14.56	3.51	3.18
OCD004	Contact 1	-6.22	0.65	-2.94	9.006	1.47	-2.82
	Contact 2	-8.22	2.49	-0.84	10.95	3	-0.43
	Contact 3	-10.28	3.87	1.65	12.99	3.97	2.27
	Contact 4	-12.45	4.64	4.58	15.05	4.35	5.42
OCD005	Contact 1	-3.15	-2.06	-8.57	6.16	-0.48	-8.97
	Contact 2	-5.53	-1.04	-6.45	8.29	0.07	-6.18
	Contact 3	-7.81	0.03	-4.32	10.36	0.709	-3.52
	Contact 4	-9.96	1.12	-2.18	12.43	1.38	-0.97
OCD006	Contact 1	-5.51	-1.34	-8.207	5.61	0.23	-7.07
	Contact 2	-7.83	0.78	-6.55	7.57	2.27	-4.87
	Contact 3	-10.01	2.49	-4.57	9.53	3.79	-2.49
	Contact 4	-12.18	3.92	-2.03	11.64	4.81	0.21
OCD007	Contact 1	-7.52	-0.8	-5.27	9.29	0.59	-5.22
	Contact 2	-9.44	1.10	-3.6	11.01	2.53	-3.49
	Contact 3	-11.36	2.44	-1.5	12.83	3.88	-1.37
	Contact 4	-13.44	3.23	1.02	14.85	4.69	1.15
OCD008	Contact 1	-11.13	2.93	-5.62	8.92	3.74	-5.87
	Contact 2	-12.51	4.35	-3.24	10.76	5.46	-3.90
	Contact 3	-13.9	5.77	-0.78	12.61	7.19	-1.87
	Contact 4	-15.31	7.19	1.73	14.51	8.88	0.17
OCD009	Contact 1	-7.33	-2.78	-5.86	9.84	-1.28	-5.14
	Contact 2	-8.94	-0.60	-4.13	11.38	0.39	-2.76
	Contact 3	-10.63	1.04	-1.91	13.06	1.35	-0.11
	Contact 4	-12.43	2.13	0.85	14.94	1.58	2.78

bioRxiv preprint doi: https://doi.org/10.64898/2026.05.11.724148; this version posted May 15, 2026. The copyright holder has placed this preprint (which was not certified by peer review) in the Public Domain. It is no longer restricted by copyright. Anyone can legally share, reuse, remix, or adapt this material for any purpose without crediting the original authors.

OCD010	Contact 1	-11.32	3.56	-5.79	8.36	3.19	-4.53
	Contact 2	-13.11	4.37	-3.11	10.14	4.03	-1.97
	Contact 3	-15.00	5.09	-0.35	12.06	4.72	0.61
	Contact 4	-16.92	5.75	2.38	14.01	5.32	3.18
OCD011	Contact 1	-7.32	-0.11	-3.42	8.67	2.35	-4.06
	Contact 2	-9.28	1.67	-1.21	10.45	4.40	-1.98
	Contact 3	-11.13	3.44	1.04	12.27	6.44	0.08
	Contact 4	-12.97	5.15	3.29	14.08	8.52	2.13
OCD012	Contact 1	-8.38	5.06	-5.03	9.07	3.73	-6.69
	Contact 2	-10.13	5.86	-2.48	10.93	4.52	-4.17
	Contact 3	-11.83	6.62	0.01	12.72	5.27	-1.63
	Contact 4	-13.52	7.3	2.44	14.54	5.91	0.91
OCD013	Contact 1	-10.23	5.06	-9.03	10.31	5.65	-8.58
	Contact 2	-12.01	6.37	-6.78	11.91	7.18	-6.41
	Contact 3	-13.79	7.65	-4.48	13.44	8.702	-4.2
	Contact 4	-15.61	8.91	-2.15	14.95	10.21	-1.94
OCD014	Contact 1	-10.97	0.38	-4.61	11.03	1.21	-3.51
	Contact 2	-12.39	1.67	-1.98	12.59	3.00	-1.31
	Contact 3	-13.75	2.89	0.67	14.22	4.77	0.92
	Contact 4	-15.16	4.01	3.36	15.96	6.52	3.13
OCD015	Contact 1	-11.17	5.31	-8.60	6.31	1.10	-8.22
	Contact 2	-12.61	6.42	-5.73	8.1	2.27	-5.69
	Contact 3	-13.97	7.57	-2.97	9.86	3.51	-3.26
	Contact 4	-15.31	8.72	-0.25	11.64	4.80	-0.90
OCD016	Contact 1	-8.94	-0.69	-2.72	5.58	-0.07	-5.3
	Contact 2	-11.05	1.42	-0.67	8.17	1.98	-2.93
	Contact 3	-13.07	2.89	1.72	10.6	3.28	-0.55
	Contact 4	-14.97	3.75	4.51	13.09	3.8	1.97
OCD017	Contact 1	6.4	-0.96	-5.1	6.4	-0.96	-5.1
	Contact 2	8.05	0.76	-3.25	8.05	0.76	-3.25
	Contact 3	9.71	1.97	-1.01	9.71	1.97	-1.01
	Contact 4	11.43	2.55	1.53	11.4	2.55	1.53
OCD018	Contact 1	-9.08	-0.69	-4.62	6.46	-0.78	-5.08
	Contact 2	-10.9	1.41	-2.97	8.33	1.65	-3.87
	Contact 3	-12.71	2.98	-0.88	10.06	3.67	-2.11
	Contact 4	-14.61	3.96	1.65	11.84	5.24	0.18

bioRxiv preprint doi: https://doi.org/10.64898/2026.05.11.724148; this version posted May 15, 2026. The copyright holder has placed this preprint (which was not certified by peer review) in the Public Domain. It is no longer restricted by copyright. Anyone can legally share, reuse, remix, or adapt this material for any purpose without crediting the original authors.

Supplemental Table 2: MNI152 coordinates of lead DBS electrodes in TRD patients

		Left Hemisphere			Right Hemisphere		
Patient ID	VC/VS Contacts	x (mm)	y (mm)	z (mm)	x (mm)	y (mm)	z (mm)
TRD001	Contact 1	-6.21	-0.85	-3.14	9.27	-0.67	-3.76
	Contact 2	-7.45	-0.29	-1.77	10.09	-0.08	-2.25
	Contact 3	-8.63	0.29	-0.34	11.18	0.56	-0.48
	Contact 4	-9.71	0.85	1.21	12.11	1.15	1.14
TRD002	Contact 1	-10.29	-3.85	-1.11	11.36	-4.22	0.4
	Contact 2	-11.6	-3.64	1.16	12.62	-3.93	2.62
	Contact 3	-13.19	-3.54	3.46	13.82	-3.61	4.82
	Contact 4	-14.77	-3.25	5.71	15.3	-3.23	7.3
TRD003	Contact 1	-4.39	0.8	-3.52	7.34	2.23	-5.33
	Contact 2	-6.68	2.09	-1.27	9.51	3.26	-3.19
	Contact 3	-9.39	3.59	1.11	11.68	4.62	-1.08
	Contact 4	-12.26	4.85	3.26	13.54	6.25	1.35
TRD004	Contact 1	-8.81	4.13	0.76	8.04	2.81	0.88
	Contact 2	-10.09	4.92	2.7	9.45	3.87	2.65
	Contact 3	-11.36	5.48	4.62	11.17	4.92	4.19
	Contact 4	-12.64	6.1	6.55	12.74	5.98	5.85
TRD005	Contact 1	-9.41	-0.72	-4.31	9.21	1.43	-1.49
	Contact 2	-10.46	0.75	-3.47	10.38	1.98	0.87
	Contact 3	-11.59	1.42	-1.11	12.79	3.57	2.88
	Contact 4	-13.78	3.25	0.91	13.95	3.82	4.05
